# Toxin MqsR cleaves single‐stranded mRNA with various 5' ends

**DOI:** 10.1002/mbo3.335

**Published:** 2016-02-05

**Authors:** Nityananda Chowdhury, Brian W. Kwan, Louise C. McGibbon, Paul Babitzke, Thomas K. Wood

**Affiliations:** ^1^ Department of Chemical EngineeringPennsylvania State UniversityUniversity ParkPennsylvania16802‐4400; ^2^ Department of Biochemistry and Molecular BiologyPennsylvania State UniversityUniversity ParkPennsylvania16802‐4400; ^3^ Center for RNA Molecular BiologyPennsylvania State UniversityUniversity ParkPennsylvania16802‐4400

**Keywords:** MqsR, mRNA, persisters, toxin/antitoxin

## Abstract

Toxin/antitoxin (TA) systems are the means by which bacterial cells become persistent; that is, those cells that are tolerant to multiple environmental stresses such as antibiotics by becoming metabolically dormant. These persister cells are responsible for recalcitrant infections. Once toxins are activated by the inactivation of antitoxins (e.g., stress‐triggered Lon degradation of the antitoxin), many toxins reduce metabolism by inhibiting translation (e.g., cleaving mRNA, reducing ATP). The MqsR/MqsA TA system of *Escherichia coli* cleaves mRNA to help the cell withstand oxidative and bile acid stress. Here, we investigated the role of secondary structure and 5′ mRNA processing on MqsR degradation of mRNA and found that MqsR cleaves only single‐stranded RNA at 5′‐GCU sites and that MqsR is equally active against RNA with 5′‐triphosphate, 5′‐monophosphate, and 5′‐hydroxyl groups.

## Introduction

Persister cells, those cells that are tolerant to antibiotics by becoming metabolically dormant, are one of the main causes of recurring infections (Fauvart et al. 2011). Since microbial infections are the leading cause of death worldwide (Rasko and Sperandio 2010), it is important to understand the mechanisms of persister cell formation and waking. The antibiotic tolerance of persister cells is not due to genetic change (Bigger 1944) but instead due to metabolic inactivity as demonstrated by both their discoverers (Hobby et al. 1942; Bigger 1944) and by subsequent experiments showing that inhibiting protein translation and ATP production converts nearly all exponentially growing cells to persister cells (Kwan et al. 2013). Persister cells may arise stochastically (Balaban et al. 2004) but are formed primarily through environmental influence (Bigger 1944; Dörr et al. 2010; Möker et al. 2010; Vega et al. 2012; Kwan et al. 2013, 2015a; Hu et al. 2015).

Persister cells become dormant through the action of toxin/antitoxin (TA) systems (Keren et al. 2004; Harrison et al. 2009; Dörr et al. 2010; Kim and Wood 2010; Luidalepp et al. 2011; Tripathi et al. 2014), and TA systems are ubiquitous in prokaryotes (Goeders and Van Melderen 2014). For example, the *Escherichia coli* genome contains at least 38 TA systems (Soo et al. 2014). Toxins are intracellular proteins that reduce metabolism in times of stress, and the neutralizing antitoxin is either protein or RNA (Wang and Wood 2011). TA systems have been classified into five (I–V) types based on the neutralization mechanism of the antitoxin. In type 1 TA systems, the antitoxin is an antisense RNA that inactivates the toxin mRNA (e.g., Hok/Sok as the first member) (Gerdes et al. 1986). In type II TA systems, the antitoxin is a protein that binds the toxin protein to inhibit it (e.g., CcdB/CcdA as the first member) (Ogura and Hiraga 1983). In type III TA systems, the RNA antitoxin binds the toxin protein to inhibit it (e.g., ToxN/ToxI as the first member) (Fineran et al. 2009). In type IV TA systems, the protein antitoxin interacts with the substrate of the protein toxin, thereby inhibiting the activity of the toxin (e.g., CbtA/CbeA as the first member) (Masuda et al. 2012). In type V TA systems, the antitoxin is an endoribonuclease that degrades specifically the toxin mRNA (e.g., GhoT/GhoS as the first member) (Wang et al. 2012).

The HipA/HipB type II system was the first TA system linked to persistence, since two point mutations in the *hipBA* operon to create the *hipA7* allele increases persistence 1000‐fold (Moyed and Bertrand 1983). Several other type II TA genes, including *yafQ*/*dinJ*,* yefM*,* relE*/*relB*, and *mazF*/*mazE* are significantly upregulated in persister cells, and production of toxins like RelE increase persistence 10–10,000‐fold (Keren et al. 2004). Similarly, production of YafQ increases the persistence of biofilm cells 10,000 fold, and its deletion decreases persisence about 2400‐fold (Harrison et al. 2009). Another type II TA toxin, MazF, induces growth arrest that results in up to a 700‐fold increase in persistence compared to a *mazF* deletion strain (Tripathi et al. 2014). In addition, the type I TA system TisB/TisA/IstR is induced by the SOS response, and TisB inceasees persistence (Dörr et al. 2009, 2010). Therefore, TA systems are intimately associated with bacterial persistence.

The MqsR/MqsA type II TA system was discovered in 2004 via a whole‐transcriptome study for its importance in biofilms (Ren et al. 2004). The structures of antitoxin MqsA and toxin MqsR were used to deduce that they are a TA system and that MqsR is a ribonuclease (RNase) (Brown et al. 2009); MqsR cuts RNA primarily at 5′‐GCU sites independent of ribosomes (Yamaguchi et al. 2009). Also, the structure of an MqsA–DNA complex, showing how MqsA binds at its target palindrome (Brown et al. 2011), was instrumental in determining that MqsA helps regulate the general stress response by controlling the sigma factor RpoS (e.g., during oxidative stress) (Wang et al. 2011) and helps to regulate biofilm formation by controlling CsgD, the regulator of curli formation (Soo and Wood 2013). Hence, in addition to controlling its own expression, MqsA functions as a global regulator by binding at other promoter positions on the chromosome. Furthermore, the MqsR/MqsA TA system controls the GhoT/GhoS TA system (Wang et al. 2012; Cheng et al. 2014) by MqsR preferentially cleaving the mRNA of antitoxin GhoS (Wang et al. 2013). Thus, a TA system was shown to control another TA system in a regulatory cascade. The physiological role of MqsR/MqsA is to help the cell withstand bile acid stress in the gastrointestinal tract (bile acid serves as an antimicrobial and generates oxidative stress conditions) (Kwan et al. 2015b). The MqsR toxin also participates in quorum sensing (González Barrios et al. 2006) and is a global regulator through varying substrate activity, which leads to differential mRNA decay (Wang and Wood 2011). Additionally, deletion of MqsR/MqsA was shown to reduce persistence (Kim and Wood 2010), and protein engineering of MqsR to make a more toxic toxin revealed paradoxically that persister cells form more readily when bacteria are less fit (Hong et al. 2012).

Along with toxins of TA systems, RNase E plays a central role in RNA processing and degradation; RNase E is a single strand‐specific endonuclease that is abundant in many bacteria including *E. coli*. RNase E in *E. coli* has a strong preference for 5′‐monophosphorylated (5′‐p) RNA as its substrate (Callaghan et al. 2005). As a result, RNase E activity is dependent on RNA pyrophosphohydrolase (RppH) which removes pyrophosphate from 5′‐triphosphorylated (5′‐ppp) primary transcripts to form mRNA with 5′‐p ends (Deana et al. 2008). Moreover, RNase E has less activity for RNA with 5′‐hydroxylated (5′‐OH) ends (Jiang and Belasco 2004).

Since MqsR has the potential to degrade nearly all mRNAs (all but 14 *E. coli* mRNAs have 5′‐GCU sites) (Yamaguchi et al. 2009), we were interested in determining how RNA secondary structure and 5′ end processing of mRNA influences MqsR degradation. We designed RNA substrates in which a single 5′‐GCU site was predicted to be single‐stranded (ssRNA), double‐stranded (dsRNA), in the loop of a stem‐loop (slRNA), or in a pseudoknot (pkRNA) and investigated their cleavage by toxin MqsR. We found that MqsR cleaves primarily ssRNA and that MqsR cleaves ssRNA irrespective of its 5′‐ppp, 5′‐p, or 5′‐OH group.

## Experimental Procedures

### *in vitro* RNA synthesis

Duplex DNA oligonucleotides containing the T7 promoter sequence were purchased (Integrated DNA Technology, Coralville, IA). Sequences of DNA templates for the ss, ds, sl, and pk RNA are shown in Table 1. RNA structures were determined using pKiss RNA prediction software (http://bibiserv2.cebitec.uni-bielefeld.de/pkiss) and structures were visualized using PseudoViewer (http://pseudoviewer.inha.ac.kr/). RNA was synthesized directly from the duplex DNA templates via *in vitro* transcription using the AmpliScribe T7‐Flash Transcription Kit (Epicentre, Madison, WI). In brief, in a standard 20 *μ*L reaction mix, 3 *μ*g of the duplex DNA templates along with transcription kit components were incubated at 37°C for 4 h. The unused DNA template (if any) was removed by adding 1 *μ*L containing 1 unit (U) of DNase I for 15 min at 37°C. The RNAs were then gel purified by fractionation through 10% denaturing polyacrylamide gels. Purified RNAs were quantified using a nanodrop spectrophotometer (Nanodrop Technologies, Inc., Wilmington, DE) and stored at −80°C.

**Table 1:** Sequence of duplex DNA templates for *in vitro* synthesis of RNAs used in this study

Name	Sequence (5′ to 3′)^a^	Length (bp)	Tm (°C)
SS‐GCU (ssRNA)	TAATACGACTCACTATAGGGAGAAAAAAAAAAAAAGCTAAAAAAAAAAAA	50	60.1
DS‐GCU (dsRNA)	TAATACGACTCACTATAGGGAGAAGGGGGCTCCCAAAAAGGGAGCCCCCA	50	71.0
SL‐GCU (slRNA)	TAATACGACTCACTATAGGGAGAAAAGGGCCGGGAGCTACCCGGCCCAAA	50	71.0
PK‐GCU (pkRNA)	TAATACGACTCACTATAGGGAGAAGGGAAACAGCCCAAAGTGCTGAAACACA	52	67.7

a Only sense strand is shown and the promoter sequence for T7 RNA polymerase is underlined.

### 5′‐end labeling of ss, ds, sl, and pk RNA with [*γ*‐^32^P] ATP

The ss, ds, sl, and pk RNA samples were radioactively labeled at their 5′ terminus using the KinaseMax 5′ End‐Labeling Kit (Life Technologies, Waltham, MA). In brief, 25 pmol of RNA was first treated with 0.1 U of calf intestine alkaline phosphatase (CIP, 0.1 U/*μ*L) for 1 h at 37°C to remove the phosphate groups (5′‐ppp) from the RNA and to create 5′‐OH substrates for the kinase reaction. The CIP was removed by a phosphatase removal reaction of the KinaseMax Kit. The 5′‐OH RNA was phosphorylated for 1 h at 37°C using 10 U of T4 polynucleotide kinase (PNK, 10 U/*μ*L) and approximately 50 pmol of [*γ*‐^32^P] ATP (7000 Ci/mmol, MP Biomedicals, Solon, OH) to generate 5′‐p RNA. The ^32^P‐labeled RNA was purified by the mini quick spin column for RNA as per manufacturer's protocol (Roche, Indianapolis, IN). The RNA concentration was determined from the CPM from a liquid scintillation counter LS 6500 (Beckman Coulter, Inc., Fullerton, CA). RNA was stored at −80°C.

### Internal labeling of 5′‐ppp, 5′‐OH, and 5′‐p ssRNA with [*α*‐^32^P] UTP

The ssRNA was radioactively labeled internally in a 20 *μ*L *in vitro* RNA synthesis reaction with 3 *μ*g of DNA, 0.33 *μ*mol/L of [*α*‐^32^P] UTP, 9 *μ*mol/L of unlabeled UTP, ATP (9 mmol/L), CTP (9 mmol/L), and GTP (9 mmol/L) along with other kit reagents for 4 h a 37°C. At this point, the primary transcripts have triphosphate at the 5′‐end (5′‐ppp). An aliquot (15 *μ*L of 445 nmol/L) was treated with CIP to convert it to 5′‐OH ssRNA. Then, an aliquot (15 *μ*L of 324 nmol/L) of this 5′‐OH ssRNA was treated with PNK and 1 mmol/L unlabeled ATP for 30 min at 37°C to make 5′‐p ssRNA internally labeled with [*α*‐^32^P] UTP.

### MqsR cleavage of RNA

For RNA labeled with [*γ*‐^32^P] ATP at the 5′‐end (5′‐p), approximately 5 × 10^−3^ pmol of RNA was used as a substrate for cleavage with 107 pmol (1×), 27 pmol (1/4×), 5.4 pmol (1/20×), and 1.1 pmol (1/100×) of purified MqsR (Brown et al. 2009) per 5 *μ*L reaction volume to determine the impact of secondary structure on MqsR cleavage. The reaction was carried out at 37°C for 6 min. For 5′‐ppp, 5′‐OH, and 5′‐p ssRNA that were internally‐labeled with [*α*‐^32^P] UTP, 0.1 pmol of each RNA was treated with 27 pmol (1/4×), 5.4 pmol (1/20×), and 1.1 pmol (1/100×) of purified MqsR and incubated for 5 min at 37°C to determine whether MqsR could cleave all three types of 5′ ends of ssRNA, and if there was any preference for these 5′ ends of ssRNA. The MqsR cleavage of RNA reaction was stopped by adding an equal volume of 2 × formamide dye (1 mol/L formamide, 20 mmol/L EDTA, 0.05% xylene cyanol, and 0.05% bromophenol blue). T1 digests with each of the labeled RNAs were prepared using 0.03 pmol of 5′‐end‐labeled RNA or 0.3 pmol of ^32^P‐UTP‐labeled RNA along with 1 or 10 U of RNase T1 (Life Technologies) and incubated at 55°C for 15 min. The reaction was stopped by adding an equal volume of 2 × formamide dye. RNA samples (MqsR or RNase T1 digested) were heated at 85°C for 3 min and fractionated through 10% denaturing polyacrylamide gels with 1 × TBE gel running buffer. RNA species were visualized with a Typhoon 9410 phosphorimager (GE Healthcare, Tyrone, PA).

## Results

**Figure 1:**
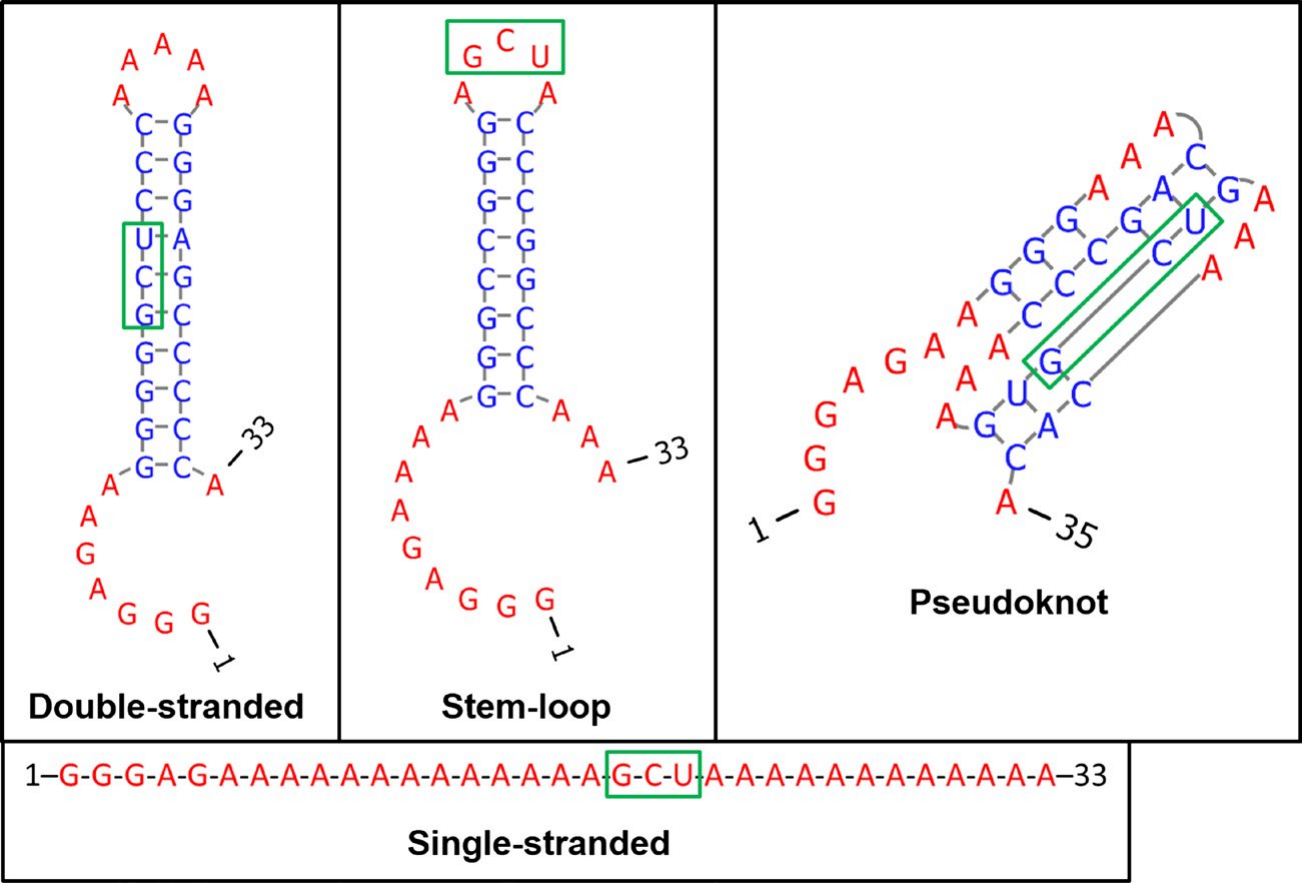
5′‐GCU sites in four different RNA secondary structures. RNA was synthesized containing a single 5′‐GCU cleavage site (boxed in green) within double‐stranded, single‐stranded, stem‐loop, and pseudoknot secondary structures.

To explore how toxin MqsR degrades mRNA, we designed four *in vitro* RNA substrates (33 to 35 nt) that contain single 5′‐GCU sites (the primary MqsR cleavage site) in ssRNA, dsRNA, slRNA, and pkRNA configurations (Fig. 1). Each of the RNAs with its GCU site was 5′‐end labeled with [*γ*‐^32^P] ATP to form RNA with 5′‐p ends. Note that MqsR cuts mRNA before and after the G nucleotide in the 5′‐GCU site (Yamaguchi et al. 2009). Since RNase T1 cleaves RNA after each G residue (Brown and Bevilacqua 2005), RNase T1 was used as a positive control that yields several RNA products for each sequence, including one that is identical to the MqsR product for each designed transcript.

**Figure 2:**
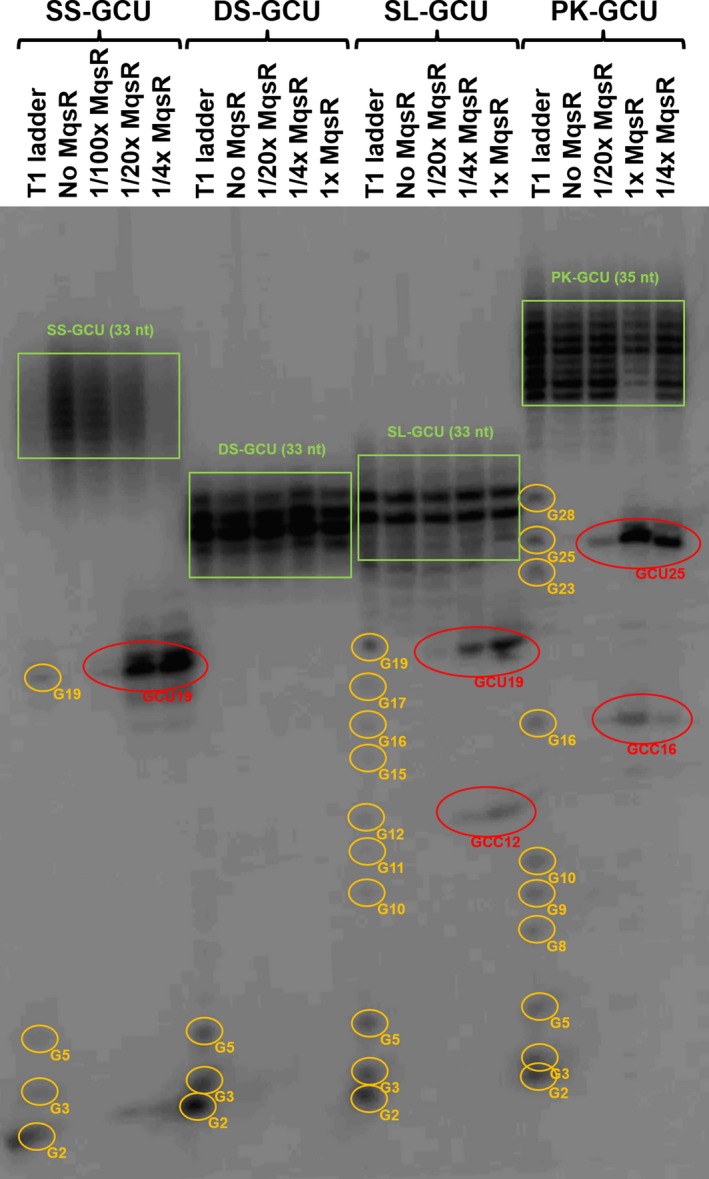
MqsR cleavage of 5′‐GCU sites in the four RNA secondary structures. In a 5 *μ*L reaction, RNA samples (0.005 pmol) were treated with 107 pmol (1x), 27 pmol (1/4x), 5.4 pmol (1/20x), and 1.1 pmol (1/100x) of MqsR for 6 min at 37°C. RNase T1 cleaves after each “G” residue, and MqsR cleaves before and after G residue of GCU sequence. Therefore, sizes of the RNA fragments were estimated via an RNase T1 digestion of each of the RNAs. The positions of cleavage at the G residue in the RNA fragments generated by RNase T1 and MqsR are indicated by the orange and red circles, respectively. Green boxes indicate transcriptional slippage.

We found that MqsR preferentially cleaves single‐stranded 5′‐GCU sites since cleavage was approximately 20‐fold higher than cleavage seen with the 5′‐GCU site in the stem‐loop and pseudoknot configuration (Fig. 2). There was no cleavage of the 5′‐GCU site in the double‐stranded RNA configuration (Fig. 2). Therefore, MqsR degradation is limited to ssRNA, and the RNA secondary structure has a large impact on enzyme activity. It should be noted that multiple bands observed in both the substrate RNAs, especially in ssRNA and its cleavage products, likely result from the phenomenon known as transcriptional slippage (Liu et al. 1994) due to the presence of mononucleotide repeats in each DNA template (Table 1). Also, there was minor cleavage at the 5′‐GCC sites for the stem‐loop and pseudoknot configurations.

**Figure 3:**
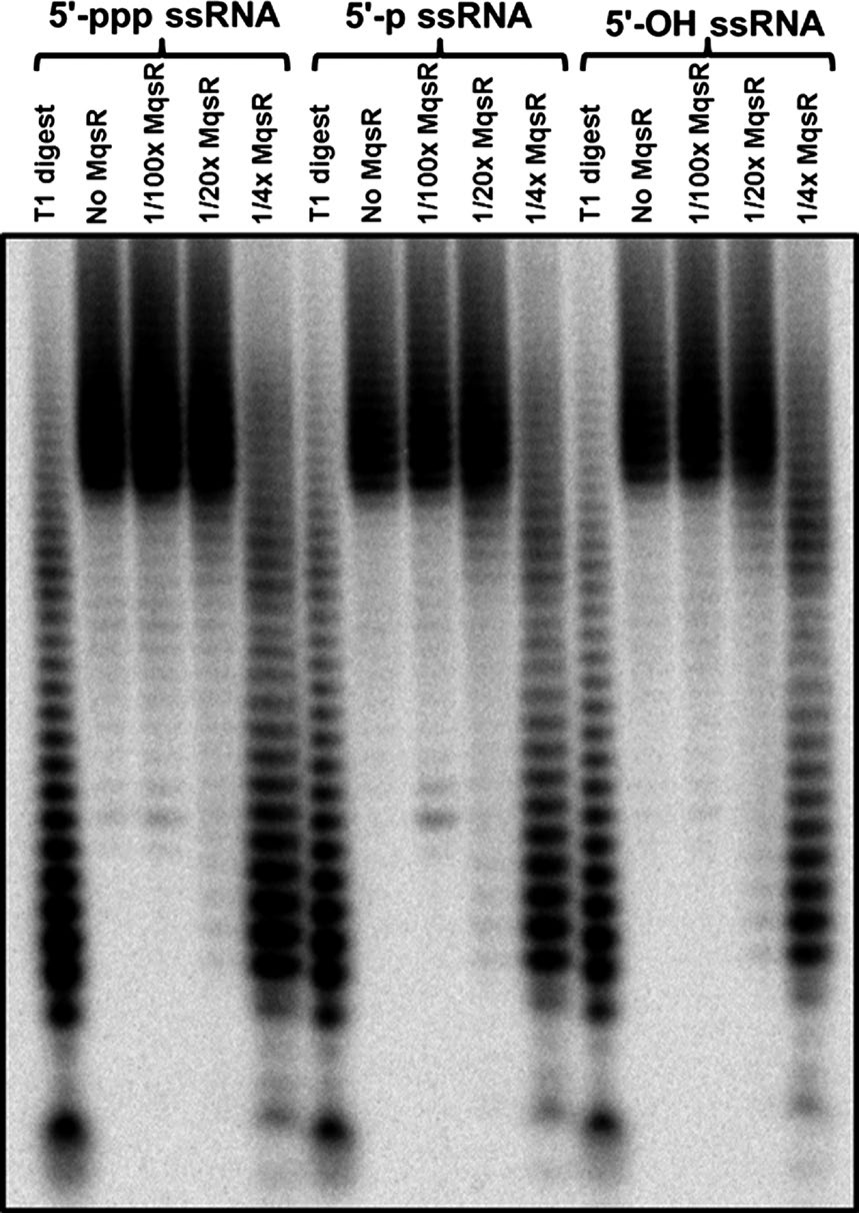
MqsR cleavage of 5′‐ppp, 5′‐p, and 5′‐OH ssRNA internally labeled with [*α*‐^32^P] UTP. Each RNA (0.1 pmol) was digested with 27 pmol (1/4×), 5.4 pmol (1/20×), and 1.1 pmol (1/100×) of MqsR for 5 min at 37°C. A ladder was prepared by digestion of each ssRNA (0.3 pmol) with 10 U of RNase T1 at 55°C for 15 min.

We also investigated whether MqsR has a preference for a 5′‐ppp, 5′‐p, or 5′‐OH ssRNA since some RNases, like RNase E, prefer 5′‐p (Callaghan et al. 2005). We synthesized 5′‐ppp, 5′‐p, or 5′‐OH ssRNA internally labeled with [*α*‐^32^P] UTP, used these ssRNAs for MqsR cleavage, and found that MqsR cleaved all three types of ssRNA (Fig. 3). Using different dilutions of MqsR and a fixed amount of each RNA, we tested whether MqsR could cleave any of them preferentially. However, the results showed similar extent of cleavage of all three types of ssRNA (Fig. 3).

## Discussion

Our results demonstrate clearly that toxin MqsR cuts primarily single‐stranded RNA. Therefore, secondary structure has a profound impact on the ability of MqsR to degrade mRNA. Similar results have been found with toxin MazF of the *E. coli* MazF/MazE TA system, which also cuts mRNA only at single‐stranded sites as shown by occluding the 5′‐ACA cleavage site with either antisense RNA or DNA (Zhang et al. 2003). Also, VapC‐mt4 toxin from *Mycobacterium tuberculosis* cuts only single‐stranded tRNA at 5′‐ACGC sequences (Cruz et al. 2015).

We also found that MqsR can degrade ssRNA independently of whether there is a 5′‐ppp, 5′‐p, or 5′‐OH end. In contrast, the primary endonuclease for degrading mRNA in *E. coli,* RNase E, has a preference for RNA containing 5′‐p ends; the 5′‐p interacts with a 5′ binding pocket on the enzyme (Callaghan et al. 2005). Physiologically, 5′‐ppp is present in primary mRNA transcripts, 5′‐p in rRNA or tRNA, and 5′‐OH in products cleaved by certain toxins such as MazF (Zhang et al. 2003; Cruz et al. 2015). While MazF and VapC‐mt4 cleave mRNA and tRNA, respectively (Zhang et al. 2003; Cruz et al. 2015), MqsR appears capable of cleaving all three types of RNA, provided that the cleavage site is not sequestered in a stable RNA duplex. This implies that MqsR is not dependent on RppH processing before it cleaves mRNA. Also, MqsR may act further on cleaved products of other toxins such as MazF to remove small RNA fragments. Moreover, evidence is accumulating that small RNA fragments (cleaved products) are associated with the stress response and other cellular functions (Cruz et al. 2015). Therefore, MqsR may work both at the primary stage to cleave its target mRNAs and rRNA/tRNAs, as well as at a secondary stage to cleave small RNA fragments. However, it remains to be determined whether MqsR can cleave rRNA/tRNAs *in vivo*.

Previous results have found that *in vivo,* MqsR targets a wide range of mRNAs related to central metabolism (González Barrios et al. 2006; Kim et al. 2010; Hong et al. 2012); these results are reasonable since MqsR needs to reduce metabolism to induce dormancy, the chief characteristic of persister cells. In effect, MqsR has the potential to degrade nearly all *E. coli* mRNAs since only 14 *E. coli* mRNAs lack the 5′‐GCU site (Yamaguchi et al. 2009); hence, MqsR is capable of degrading nearly all mRNA to stop translation and reduce cell growth. In addition, of the 14 mRNAs that lack 5′‐GCU sites, the transcript of toxin GhoT is not cleaved by MqsR which leads to induction of another TA system, GhoT/GhoS, which further increases persistence by reducing ATP levels through membrane damage (Wang et al. 2012, 2013; Cheng et al. 2014). Therefore, the results here demonstrate that other forms of RNA are suitable targets for MqsR which indicates that this enzyme may inhibit translation by additional mechanisms to halt metabolism and create persister cells.

## Conflict of Interest

The authors declare no conflicts of interest.
